# An open-source software for building and simulating ordinary differential equation models in biology

**DOI:** 10.1371/journal.pone.0329148

**Published:** 2025-08-26

**Authors:** Brenno Lemos Melquiades dos Santos, Diego Augusto Silva Castro, Sávio Francisco Cirino da Paz, Davi Jannotti Coelho Pinheiro, Bárbara de Melo Quintela, Marcelo Lobosco, Alexandre Bittencourt Pigozzo

**Affiliations:** 1 Department of Computer Science, Federal University of São João del-Rei, João del-Rei, Minas Gerais, Brazil; 2 Graduate Program in Computational Modeling, Federal University of Juiz de Fora, Juiz de Fora, Brazil; Ariel University, UNITED KINGDOM OF GREAT BRITAIN AND NORTHERN IRELAND

## Abstract

Mathematical and computational modeling are transforming biological research by enabling detailed exploration of complex systems. However, building computational models of biological phenomena often demands expertise in mathematical modeling and programming, creating barriers for researchers without such backgrounds. Existing software tools for computational modeling are frequently complex, overly general, or proprietary, limiting their accessibility and usability. To overcome these challenges, we present *ODE-Designer*, an open-source software tool that facilitates the construction and simulation of Ordinary Differential Equation (ODE) models in biology. A central feature of *ODE-Designer* is its intuitive visual interface, designed to be user-friendly and accessible. The software includes a graphical user interface with a node-based editor that allows users to create models without writing code. It supports model simulation and automatically generates the corresponding code, enabling efficient model exploration and aiding students in understanding core modeling principles. We propose that *ODE-Designer* serves as a valuable resource for both research and education in computational biology, improving accessibility and promoting a quantitative perspective in biological research. The software is freely available at: https://github.com/ufsj-dcomp/ode-designer-rs/.

## Introduction

Mathematical and computational modeling have become increasingly vital in modern scientific research, driven by the need for rapid, comprehensive understanding of complex systems across diverse disciplines. Computational models complement experimental approaches by enabling investigations at scales that are difficult or impossible to study using traditional methods alone [1]. These models allow researchers to explore hypothetical scenarios, make predictions, and test new hypotheses, thereby accelerating scientific discovery. In cellular biology, for example, the inherent complexity of biological processes—arising from numerous interacting components across multiple spatial and temporal scales—has intensified the need for mathematical modeling [2]. Such models enable simulations and *in silico* experiments that are considerably faster than traditional *in vitro* or *in vivo* methods, often offering greater spatiotemporal resolution [2]. As a result, computational modeling has become an indispensable tool for exploring both biological phenomena and physical systems.

Developing robust computational models involves several critical steps, including clearly defining objectives, validating model accuracy, and refining models through iterative cycles. Among these, model implementation is often particularly challenging, as it requires proficiency in numerical methods, programming, data structures, and specialized software libraries. Errors at this stage can compromise the validity and reproducibility of the entire modeling effort.

To address these challenges, we present *ODE-Designer*, an open-source software tool designed to simplify the implementation and simulation of models based on Ordinary Differential Equations (ODEs). Our primary aim is to provide a visual modeling platform that lowers technical barriers to entry, enabling researchers, particularly those without programming expertise, to construct and simulate computational models more easily. By reducing the time and effort required for implementation, *ODE-Designer* allows users to concentrate on scientific inquiry and model analysis.

*ODE-Designer* features a user-friendly graphical interface that enables users to construct ODE models by visually assembling components in a node-based editor. The software then automatically generates the code required for model simulation and result visualization. Key contributions of this work include: a) an intuitive graphical representation of mathematical models based on ODEs; b) a template-based code generator that automates the creation of implementation code; and c) the ability to develop and simulate models without writing code, while still providing access to the generated code for further extension and refinement.

Beyond its applications in research, *ODE-Designer* was also developed with education in mind. Its intuitive interface, real-time simulation feedback, and code-free modeling environment make it well-suited for classroom use and exploratory learning. By enabling hands-on construction and immediate visualization of ODE-based systems, the platform supports active learning and constructivist pedagogical approaches, fostering conceptual understanding of dynamic processes in biology, physics, and engineering.

## Related work

In this section, we review existing tools for computational modeling and compare their functionalities with those of the proposed software.

Snoopy [3] is a tool designed for constructing, animating, and simulating Petri nets, including stochastic and colored variants that extend the classical framework. Models are represented as directed graphs composed of two node types—places and transitions—and four edge types: stochastic, immediate, deterministic, and planned. Each edge type conveys distinct semantics, with places representing populations and transitions capturing events that alter these populations. However, Snoopy does not natively support the direct construction or simulation of Ordinary Differential Equations (ODEs).

InsightMaker [4] is a web-based modeling and simulation environment that allows users to build computational models using an interactive graphical interface. It supports both System Dynamics and agent-based modeling paradigms, enabling the creation of models on a visual canvas. InsightMaker internally converts System Dynamics diagrams into ODEs and offers several numerical solvers, including the Euler and fourth-order Runge–Kutta methods. Despite its support for ODE-based simulation, the platform lacks a graphical interface specifically designed for constructing ODE models.

Virtual Cell (VCell) [5] is a web-based platform for modeling cellular biological systems, organized around a central database and based on a rule-driven modeling framework. VCell supports a wide range of simulation methodologies, including deterministic simulations (compartmental ODEs and partial differential equations), stochastic simulations (via stochastic simulation algorithms), spatial stochastic simulations (using tools like Smoldyn), and hybrid deterministic/stochastic approaches. It also includes support for agent-based modeling. These features enable the simulation of complex biological phenomena such as metabolic processes, electrophysiology, membrane dynamics, and lateral diffusion. However, its scope—focused specifically on cellular biological systems—and its modeling complexity may present a steeper learning curve for users interested primarily in general-purpose ODE-based modeling.

CompuCell3D [6] is an open-source framework designed for simulating multicellular and virtual tissue dynamics. Based on the Cellular Potts Model (CPM), it employs a modified Metropolis algorithm within the Monte Carlo method to iteratively minimize the system’s Hamiltonian. This allows simulation of biological processes such as tissue development, homeostasis, and disease progression. CompuCell3D supports modeling of various cellular behaviors, including movement, adhesion, growth, mitosis, and chemotaxis. Nonetheless, it is primarily tailored to spatial modeling using energy functions and partial differential equations and does not offer direct support for ODE-based model construction and simulation.

*ODE-Designer* sets itself apart by providing native support for the visual construction and simulation of ODE models. Its graphical interface allows users to build models component by component while simultaneously accessing the automatically generated implementation code. This step-by-step approach supports both model development and verification. Unlike tools such as VCell and CompuCell3D, which are optimized for specific biological applications, *ODE-Designer* is domain-agnostic and can be used across a wide range of scientific and engineering disciplines where ODE-based modeling is relevant.

Moreover, while many existing modeling platforms prioritize research-grade simulation capabilities, few are explicitly designed with educational usability in mind. *ODE-Designer* addresses this gap by offering a user experience tailored to both teaching and exploratory model development, making it an effective tool for learning and instruction as well as research.

## Materials and methods

**Fig 1:**
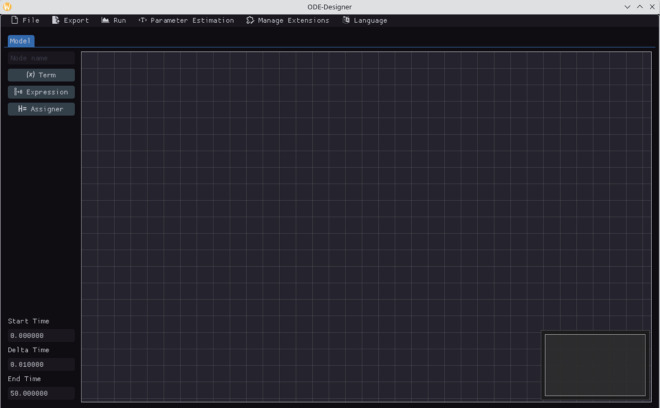
ODE-Designer initial screen showing the menu at the top, the model building tab with the Node-Based Editor on the right side and a sidebar menu on the left side. The sidebar has options for creating nodes and for setting the values for the start time, time step and the end time.

The software developed in this work features a GUI that utilizes a node-based editor, as illustrated in Fig 1. The nodes represent abstractions of commonly used components in ODEs, such as constants, variables, and mathematical expressions. Details regarding the specific node types offered within the GUI will be presented throughout this section.

*ODE-Designer* implementation is divided into two core modules: a) the GUI module and b) the intermediate representation (IR) module. The GUI module handles window rendering, node editor actions and events, user interactions, and plot management. The IR module focuses on defining the JSON representation of models, saving and loading models, and generating Python code using templates. The codebase of *ODE-Designer* is designed to be modular, easy to maintain, and extendable. The software architecture is organized in the following main submodules:

- Application submodule: Responsible for rendering the node editor and its components, managing global state, and controlling auxiliary windows (plotting tabs, extension manager, context menu).
- Nodes submodule: Manages the logic behind different node types, including how they are drawn in the GUI. It provides methods for receiving and sending messages to notify state changes and trigger necessary GUI updates.
- Extension submodule: Implements structures for parsing and loading custom node definitions (Python functions).
- Expression submodule: Defines a tree structure representing the mathematical model’s expressions. It also provides structures used during message passing to communicate partial model changes.
- Messaging submodule: Implements the communication structure used by other modules within the application.

*ODE-Designer* is implemented in the Rust programming language [7] (www.rust-lang.org/). Rust was chosen due to its performance, comparable to C++, and its rich ecosystem of libraries that simplify model serialization and template-based code generation.

### Model construction

Model construction in *ODE-Designer* involves creating nodes and establishing connections between them. These connections are made through pins, which come in two types: a) input pins and b) output pins. Input pins receive values from other nodes, while output pins transmit values. Connections are always unidirectional, linking an output pin from a source node to an input pin on a destination node.

Expressions are constructed by combining Terms and other expressions within the software. Terms represents variables, parameters or constants. An Assigner node holds a single expression, representing the right-hand side of an ODE. The name Assigner denotes a node that receives an expression and assigns it to a target variable. The input expression is a right-hand side of an ODE.

Once a model is constructed, users can interact with the software in multiple ways: they can execute simulations directly within the GUI, generate PDF reports containing the simulation results, or export the corresponding Python code representation. Both GUI-based simulations and those executed via exported Python code leverage the solve_ivp method from the scientific computing library Scipy [8].

### A visual representation for ODEs

Many tools for simulating ODE models still require users to write code or understand formal equation syntax, which can present a barrier to entry for beginners and limit accessibility. To address this, we propose a structured visual representation that enables users to define and understand models through an intuitive graphical interface. This visual syntax eliminates the need for code and makes the structure of a model immediately apparent, supporting both novice users and experts seeking clarity.

ODEs are governed by fundamental principles, such as the Mass Action Law [9], which states that “the number of interactions between two particles depends on the concentration of both”. In other words, a higher concentration of reactants increases the probability of interaction, provided the necessary conditions are met. To illustrate how the interaction between species can be visually structured and mathematically encoded in ODE-Designer, we use the classic SIR (Susceptible-Infectious-Recovered, see Eq. (1)) model from epidemiology. In this model, susceptible individuals (S) may become infected (I) through contact with infectious individuals, while infected individuals eventually recover (R). The rate of new infections follows a mass-action-like interaction between S and I: the more individuals in each group, the higher the probability that they come into contact, leading to a transition from S to I:

$$\frac{dS}{dt}=-b_{d}SI,\;\frac{dI}{dt}=b_{d}SI-gI,\;\frac{dR}{dt}=gI,$$ (1)

where:

- *S*(*t*) is the number of susceptible individuals at time *t*,
- *I*(*t*) is the number of infectious individuals,
- *R*(*t*) is the number of recovered individuals,
- *b*_*d*_ is the infection rate coefficient,
- *g* is the recovery rate coefficient.

ODE modeling involves defining expressions that capture interactions between populations and their environment. This concept is visually represented in the software’s node-based editor, where nodes and their connections depict model components. This is naturally represented in ODE-Designer using directed edges (pins), expressions and assigners that encode such interaction terms.

**Fig 2:**
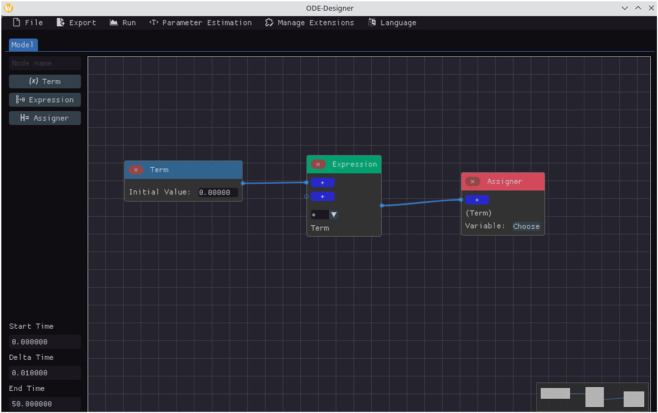
Types of nodes available in ODE-Designer. The nodes colored blue are the nodes called Terms. Terms are used to define the variables and parameters of the model. The nodes colored green are the Expression nodes. The expressions combine terms and other expressions to form the ODEs of the model. The nodes colored red are the Assigner nodes. The Assigner node assign an equation as the right-hand side of the ODE of a model variable.

Table 1 lists the node types available in ODE-Designer along with their corresponding functions. These node types are also visually represented in the graphical user interface, as shown in Fig 2.

**Table 1:** Node types available in ODE-Designer.

Node Type	Represents	Example of Use
Term	Variables, parameters and constants.	The expression *b*_*d*_*SI* includes the parameter *b*_*d*_ and the variables *S* and *I*.
Expression	Any mathematical expression used in the system’s equations.	In the SIR model, the expressions include: 1) *b*_*d*_*SI*; 2) *gI*; and 3) *b*_*d*_*SI*−*gI* (combining 1 and 2).
Assigner	A special node that assigns an expression, representing the right-hand side of an ODE, to a variable.	The expression *b*_*d*_*SI* − *gI* can be assigned to an Assigner node linked to the variable *I*.

### Pin and node relationships

The arrangement of pins on nodes is designed for intuitive interaction, easing the learning curve for users. The relationship between node types and pin configurations is as follows:

- **Term node:** One output pin, no input pins (initial nodes).
- **Expression node:** *N* input pins, one output pin.
- **Assigner node:** One input pin, no output pins (terminal nodes).

Thus, terms act as initial nodes, as they do not require input connections. Expressions can be linked to other expressions or assigner nodes, while assigner nodes function as terminal nodes.

*ODE-Designer* supports the modeling of both linear and nonlinear ODEs by combining variables and expressions using standard mathematical operators (+, –, *, and /). Additional functions, such as exponential, square root, and trigonometric operations, can be incorporated via extension nodes.

### Code generation and interactive simulation

An intermediate representation (IR) was defined to facilitate the saving, loading, and generation of implementation code for the models. The JSON format was selected for its widespread use and well-defined structure, supporting data types such as lists and hash tables, which simplifies software development. For example, the JSON representation of the extended SIR model (presented in Results Section) can be found in the Supporting Information. The IR, along with a template file, serve as inputs to the code generator.

The code generation process extracts model information from the IR and injects it into placeholders within the template using string substitution. The template file contains the essential code for numerically solving, simulating, and plotting the model results. It also includes necessary libraries, such as numpy, scipy, and matplotlib. These templates are designed for both clarity and brevity, making them useful as educational material in addition to their practical purpose. Listing 1 presents a portion of the Jinja template responsible for extracting values of variables and constants to solve the ODE system. This snippet demonstrates the use of control structures like conditionals (if) and loops (for).

**def** system(t: np.float64, y: np.ndarray, *constants) -> np.ndarray:

    {% **for** arg in populations -%}

    {{- arg.name },

    {%- endfor % = y

    {%- **if** constants %}

    {% **for** arg in constants -%}

    {{- arg.name },

    {%- endfor % = constants

    {% endif -%

    # ...

**Listing 1.** Jinja template snippet.

The generated code serves two purposes: a) as an export option for users, allowing them to export the model as Python code, and b) for internal simulation, enabling the software to use the generated code to simulate the model and display results within the GUI. This approach ensures that the plots displayed within the software and those generated by the exported code are identical.

**Fig 3:**
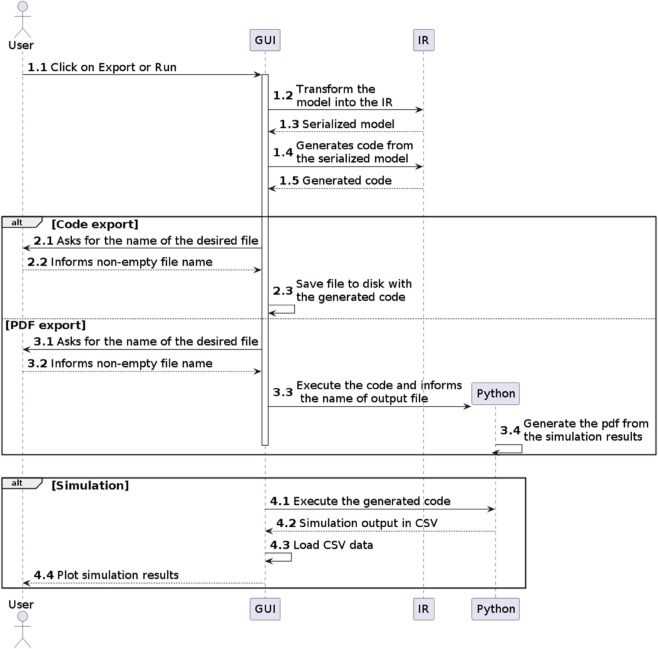
Sequence diagram representing the processes of code export, PDF export, and interactive simulation. The diagram shows the main interactions between the user, the GUI and the code generator called the IR (intermediate representation) module, after the user chooses to simulate the model interactively or one of the export options.

Fig 3 illustrates a sequence diagram showing some of the interactions between the user and the software. It outlines the sequence of events triggered when the user clicks “Export” or “Run” in the menu, highlighting the interaction between the GUI and IR modules.

*ODE-Designer* also supports the creation of custom nodes by users, referred to as extension nodes. Further details are provided in the next section. A discussion on the distribution of *ODE-Designer* is presented in Section Software distribution.

### Software extensibility

Software applications rarely meet all conceivable user needs, and *ODE-Designer* is no exception. Anticipating every required functionality for constructing ODE systems is challenging. To enhance flexibility, *ODE-Designer* incorporates an extension system that allows users to define custom nodes.

Beyond the standard nodes for ODE construction, users can develop and integrate extensions to perform additional operations not available through the GUI. These extensions are written in Python and introduce new node types into the interface.

Functions decorated with @node in an extension file are interpreted as nodes, with their function names serving as identifiers in the GUI. The number of function parameters determines the number of input pins. By default, these nodes display expressions in standard function notation (*e.g.*, *function(param1, param2, ...)*), but this can be customized using the *format* parameter in the decorator.

Listing 2 provides an example extension defining nodes for computing the sine function and exponentiation. Once imported into the GUI, these extension nodes become available as new expression nodes with the purple color.

Since these nodes are defined in Python, they integrate seamlessly into the simulation process without affecting model execution or visualization. The generated code includes the extension functions directly, ensuring consistency. Additionally, models saved by the interface retain references to their associated extension files, enabling automatic loading of necessary extensions when reopening a model. This feature facilitates collaboration, allowing users to share and reuse custom extensions.

**import** math

    @node

**def** sin(x):

**return** math.sin(x)

    @node( **format**=’$1 ^∧^ $2’)

**def pow**(x, y):

**return** x ** y

**Listing 2.** Python code defining extension nodes.

### Workflow for model building and simulation

Upon launching *ODE-Designer*, the initial interface presents an empty node editor along with a side menu for creating different node types as shown in Fig 1. Nodes can be added to the editor either via this menu or through the context menu accessed by right-clicking.

Model building in *ODE-Designer* follows a structured workflow:

- **Creating Term Nodes:** The first step involves defining term nodes for each variable, parameter, and constant in the model.
- **Constructing Expression Nodes:** Expression nodes define mathematical operations and interaction terms. These nodes progressively build up the full right-hand side expressions for each ODE.
- **Assigning ODEs to Variables:** Finally, assigner nodes associate each constructed expression with its corresponding variable, completing the model definition.

Once the model is constructed, users specify simulation parameters, including initial time, time step (temporal discretization), and simulation duration, via the sidebar. Optional settings allow users to label the *x*- and *y*-axes for visualization.

When a model is executed, *ODE-Designer* automatically generates Python code (available in the Supporting Information), leveraging the SciPy library to numerically solve the ODE system. Simulation results are stored in a CSV file and visualized within the GUI using the ImPlot library (github.com/epezent/implot).

Upon simulation completion, *ODE-Designer* opens new tabs to display the results. The first tab presents a combined population plot, while subsequent tabs provide individual population dynamics. The tab-based interface enables seamless switching between model construction, simulation results, and additional functionalities such as parameter estimation.

### Software distribution

Software distribution is inherently complex, with no universal solution. Different distribution strategies offer trade-offs that impact software accessibility and ease of installation. Developers targeting experienced programmers may rely on users having the necessary tools (compilers, package managers), while broader audiences require user-friendly deployment methods.

Our goal was to distribute *ODE-Designer* as a single, portable executable. However, the software depends on external packages, including a Python interpreter and the scipy, numpy, and matplotlib libraries. To ensure smooth installation, we bundle these dependencies alongside the software, allowing version control independent of the user’s system configuration.

Linux distributions utilize a variety of package managers and formats, making universal distribution challenging. Maintaining multiple versions compiled against different dependencies would require extensive testing and frequent updates. To simplify this process, we adopted AppImage (appimage.org/), a format introduced in 2004 that enables software to run on various Linux distributions without modification.

An AppImage file encapsulates a Linux directory structure and multiple dependencies. When executed, it unpacks into RAM as a virtual file system, with a designated entry point for launching the application. The AppImage distribution of *ODE-Designer* includes a pre-configured Python interpreter and all necessary libraries (scipy, numpy, matplotlib), ensuring compatibility across different Linux environments.

A version release management tool was developed using Docker [10]. The tool runs within a Debian-based container (2019 version) pre-configured with all required dependencies, enabling cross-platform compilation for both Linux and Windows.

Unlike Linux, Windows lacks a native file format that bundles multiple executables into a single package. As a result, Windows software is typically distributed as compressed archives (*e.g.*, ZIP files) or installers that extract files into a structured directory. Consequently, the Windows version of *ODE-Designer* is provided as a compressed folder containing its required dynamic dependencies and a portable Python interpreter preloaded with scipy, numpy, and matplotlib.

## Results

To demonstrate the capabilities of *ODE-Designer*, we constructed and simulated two mathematical models: a) a variation of the classic Susceptible-Infected-Recovered (SIR) model; and b) a simplified model of immune response regulation.

### Extended SIR model

The model used in this example is based on the SIR framework introduced earlier but includes an additional variable representing the concentration of pathogens in the environment. This formulation is based on the work by Handel et al. [11] and does not correspond to the classical SEIR model, in which the “E” compartment refers to individuals in a latent period between infection and becoming infectious. In contrast, here the variable *E* account for pathogen presence in the environment. For this reason, we refer to it as an “extended SIR model” rather than an SEIR model.

In this model, susceptible individuals become infected either by direct contact with infected hosts (at rate *b*_*d*_) or through environmental exposure to the pathogen (at rate *b*_*e*_). Infected hosts contribute to the environmental pathogen load at rate *p*, while the pathogen decays at rate *c*. Additionally, new individuals are born susceptible at rate *b*, and all individuals have a natural death rate *n*. The full set of ODEs is given in Eq 2:

$$\frac{dS}{dt}=b-b_{d}SI-b_{e}SE-nS,\;\frac{dI}{dt}=b_{d}SI+b_{e}SE-gI-nI,\;\frac{dR}{dt}=gI-nR,\;\frac{dE}{dt}=pI-cE.$$ (2)

**Fig 4:**
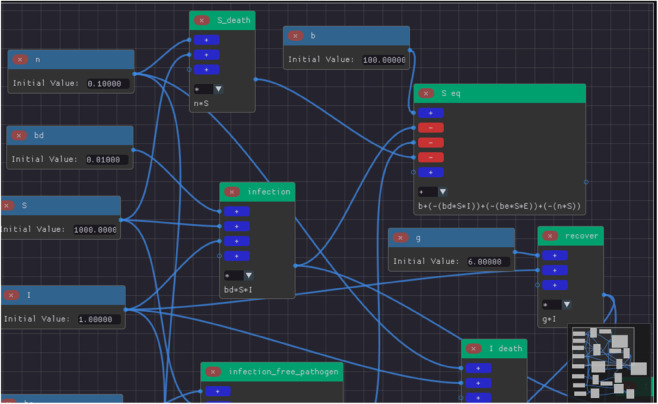
Extended SIR model built on ODE-Designer highlighting some terms (blue nodes) and expressions (green) nodes. We can observe the expressions for the death and infection of susceptible (*S death* and *infection*), recovery (*recover*) and the final equation of susceptible population (*S eq*).

**Fig 5:**
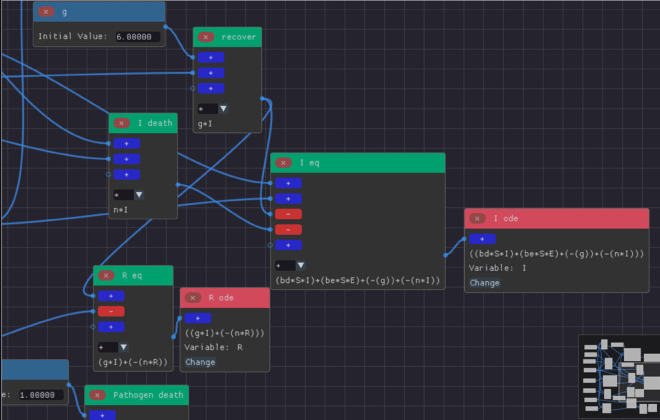
Extended SIR model built on ODE-Designer highlighting some terms, expressions and assigner nodes. The assigner nodes are the red ones. The assigner nodes for the *I* and *R* populations are shown as the *I ode* and *R ode* nodes respectively. We can observe that the inputs of the assigner nodes are expression nodes containing the full equations that will form the right-hand sides of the ODEs.

Based on the workflow described in previous section, we can build the extended SIR model on ODE-Designer by creating the terms, expressions and finally the assigner nodes:

- **Term Nodes:** Creation of term nodes for the variables *S*, *I*, *R*, *E* and all parameters of the model (represented as blue nodes in Fig 4).
- **Expression Nodes:** Expressions represented as the green nodes are built by combining the terms and other expressions as shown in Figs 4 and 5.
- **Assigner Nodes:** Finally, an assigner node is constructed for each variable of the model and it defines the right-hand side of that variable’s ODE (see S_ode and R_ode in Fig 5).

**Fig 6:**
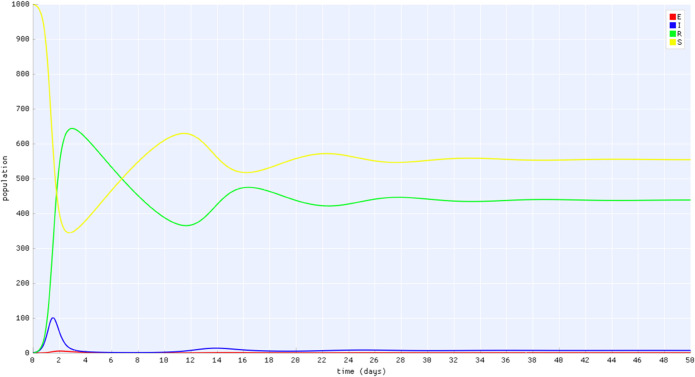
View of the simulation results on ODE-Designer GUI: populations of S (yellow), I (blue), R (green), and E (red) over time.

After the initial condition and parameter values are defined, the model can be simulated through the GUI by clicking the Run option in the menu. In Fig 6, an example of simulation result is presented where we observe that, after an initial period of disease transmission and a transitory oscillatory dynamics, the populations tend to equilibrium.

### Immune system regulation model

We also implemented a simplified model of innate immune response regulation following skin tissue injury. This model tracks the dynamics of:

- Neutrophils (*N*),
- Regulatory Macrophages (*M*_*reg*_),
- Pro-inflammatory Cytokines (*CH*),
- Anti-inflammatory Cytokines (*AC*), and
- Tissue Damage (*TD*).

The model is defined by the following set of ODEs (Eq (3)):

$$\begin{array}{c} \frac{dN}{dt}=-\beta_{N}N-m_{N}N+\frac{s_{N}(TD+C)}{\left(1+\alpha AC\right)},\\ \frac{dM_{reg}}{dt}=\gamma N-m_{M_{reg}}Mreg,\\ \frac{dCH}{dt}=\frac{\beta_{C}N}{\left(1+\alpha AC\right)}-m_{C}C,\\ \frac{dAC}{dt}=\beta_{AC}M_{reg}-m_{AC}AC,\\ \frac{dTD}{dt}=\beta_{N}N-kM_{reg}TD \end{array}$$ (3)

**Fig 7:**
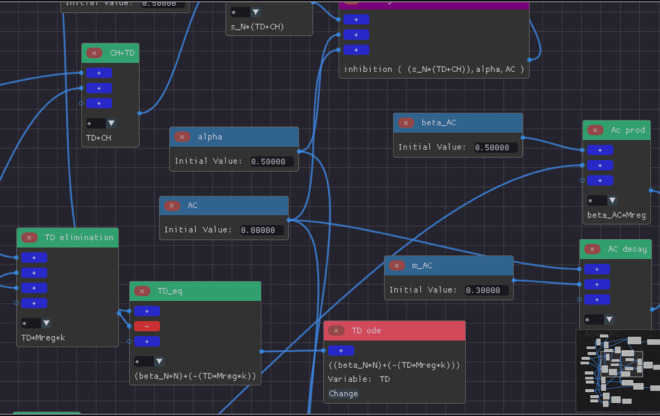
Immune response regulation model built on ODE-Designer highlighting some terms, expressions and assigner nodes. We can observe expressions for neutrophil migration using the extension node (purple node), tissue damage elimination (*TD elimination* node) and the complete equation for the tissue damage (*TD_eq* node). Besides, we can also observe the assigner node that defines the ODE for the TD population (*TD ode* node).

**Fig 8:**
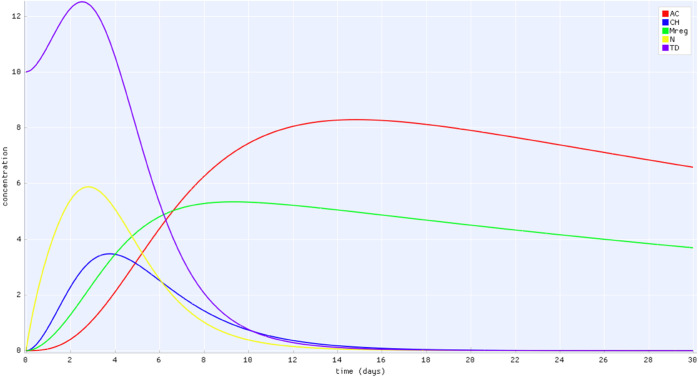
View of the simulation results on ODE-Designer GUI. The results shows the immune response regulatory control over inflammation. The populations are: *AC* (red), *C* (blue), *M*_*reg*_ (green), *N* (yellow) and *TD* (purple).

The model emphasizes the role of anti-inflammatory regulation in preventing chronic inflammation. The anti-inflammatory response is mediated by regulatory macrophages and anti-inflammatory cytokines. Without regulatory macrophages, tissue damage and inflammatory mediators grow unchecked. Similarly, an insufficient anti-inflammatory response can lead to recurring inflammation cycles, even after an initial resolution (results not shown). Fig 7 presents the model constructed using ODE-Designer, while Fig 8 displays a simulation result in which inflammation is effectively regulated by the anti-inflammatory response mediated by *M*_*reg*_ cells.

As discussed in the next section, ongoing enhancements aim to streamline the construction of complex models, including user-defined expression nodes to improve modularity. Additional example models can be found in the examples directory and a tutorial video showing the building and simulation of the predator-prey model can be found in the tutorial directory of the code repository.

## Discussion

A key advantage of *ODE-Designer* is its visual representation of mathematical models, which greatly facilitates equation construction and comprehension. This graphical approach enables users to clearly track the development of mathematical expressions and their integration into the ODE system. Additionally, users can easily inspect the inputs, operations, and signal flow within each expression, thereby improving their understanding of the model’s underlying logic.

A central advantage of *ODE-Designer* is its suitability for teaching and learning scenarios. By providing a unified environment for model construction, simulation, and analysis—without requiring coding or advanced training—the tool promotes active learning and lowers the barrier to entry for computational modeling. This makes it particularly valuable in undergraduate and graduate courses covering systems biology, pharmacokinetics, ecology, or any area where ODEs play a foundational role.

The platform supports inquiry-based and constructivist learning, allowing students to iteratively manipulate model parameters, test hypotheses, and visualize outcomes. Such real-time feedback facilitates the development of modeling intuition and strengthens the connection between mathematical formulations and system behavior. Its lightweight design and ease of deployment also make it ideal for use in online courses, workshops, and interdisciplinary curricula.

In this context, *ODE-Designer* fills a critical gap in the ecosystem of modeling tools by focusing on clarity, interactivity, and educational value. It thus complements more complex platforms oriented toward advanced simulation tasks.

For researchers, *ODE-Designer* streamlines the development of models of low to moderate complexity, significantly reducing implementation time and allowing for a greater focus on hypothesis exploration and result analysis.

The correctness of software-generated implementations can be verified using multiple approaches. Users can directly access and inspect the generated code, comparing simulation results with alternative implementations. Additionally, for models with known analytical solutions, numerical results can be validated against exact solutions.

Some of the techniques employed in *ODE-Designer*, such as template-based code generation, may also be applicable to other domains. For instance, they could be leveraged in computational biology simulators, allowing users to define high-level model representations that are then translated into executable code.

One challenge in visual representation is that models with a large number of nodes and connections can become difficult to interpret. Overlapping links may create visual clutter, reducing readability. A potential solution is the introduction of a new node type that allows users to define complex expressions, thereby reducing the number of required expression nodes. Additionally, enabling users to create custom nodes by combining existing expressions could further enhance usability.

In its current version, *ODE-Designer* supports a limited but commonly used set of mathematical operations directly through its GUI. This design choice reflects the project’s educational and accessibility goals. Nevertheless, we acknowledge that this may restrict more advanced users who require non-standard or domain-specific operations. To address this limitation, the software includes a plugin architecture that allows users to write custom extensions in Python. These extensions can define new node types or functional behaviors, which are automatically incorporated into the interface and used like native components. This extensibility provides a flexible pathway for customizing the tool for specific research needs, while preserving the benefits of a visual modeling environment. Additionally, although the graphical interface does not currently support direct export of simulation results to CSV, the Python code generated by *ODE-Designer* produces output arrays that can be easily saved to CSV or integrated into custom post-processing pipelines.

While the tool is currently focused on ODE-based models, future development will explore support for broader modeling paradigms. One potential direction involves the integration of *ODE-Designer* with tools such as VisualPDE [12], which enables interactive simulation and visualization of partial differential equations in the browser. Such integration would further expand the scope of the platform and provide a bridge between intuitive model construction and advanced simulation capabilities.

A usability study is planned to evaluate the GUI and overall user experience. This analysis will be conducted in undergraduate and postgraduate courses related to computational modeling. Initial feedback from some students has already resulted in improved error handling, for example, in cases such as division by zero.

Overall, *ODE-Designer* provides an effective and extensible platform for building, simulating, and analyzing dynamical systems. Its emphasis on usability, educational value, and workflow clarity makes it a valuable complement to more comprehensive modeling environments, particularly in scenarios where simplicity, transparency, and interactivity are priorities.

## Conclusion

This work presented the development of *ODE-Designer*, a software tool designed to automate the implementation and simulation of ODE-based computational models. Through its graphical user interface (GUI), *ODE-Designer* enables users to visually conceptualize mathematical models, automatically generating the necessary code for implementation, simulation, and result visualization.

The source code is available at https://github.com/ufsj-dcomp/ode-designer-rs/, with releases accessible at https://github.com/ufsj-dcomp/ode-designer-rs/releases. *ODE-Designer* is compatible with Linux and Windows and requires Rust, Python 3.11 or higher, and the numpy, scipy, and matplotlib libraries.

Several avenues exist for extending *ODE-Designer*’s capabilities:

- **Expanded Code Generation:** Introducing code generation in Rust and enabling real-time, interactive ODE simulations.
- **Stochastic Model Support:** Integrating the Gillespie algorithm for stochastic simulations while maintaining the node-based editor and creating dedicated templates for stochastic models.
- **Parameter Estimation:** Implementing functionalities for loading experimental data, selecting parameters for adjustment, and visualizing results.
- **Sensitivity Analysis:** Extending the software to include parameter sensitivity analysis tools and corresponding visualization methods.
- **Partial Differential Equations (PDEs):** Adding support for PDEs by introducing nodes representing diffusion, chemotaxis, and other relevant operators, along with corresponding templates. We also plan to integrate the platform with existing tools such as VisualPDE.
- **Web-Based Version:** Developing an online version of *ODE-Designer* using Rust’s WebAssembly support to increase accessibility.
- **Improved Data Import/Export:** Supporting additional file formats widely used in computational biology, such as CellML [13].

*ODE-Designer* is under active development. Current efforts include:

- **Language Localization:** Support for English and Brazilian Portuguese.
- **Enhanced Error Handling:** A notification system for informative error messages using modal windows.
- **Parameter Estimation Module:** An integrated extension for parameter estimation.
- **Stochastic Simulation Library:** Development of an optimized Rust library for simulating stochastic models using the first reaction method. Future versions of *ODE-Designer* will generate code compatible with this library.

We believe that with continuous development efforts to introduce the functionalities mentioned before, ODE-Designer can have a wider and long-term utility.

## Supporting information

S1 Data Supporting information. (ZIP)
