# Supplementary material for: An open-source software for building and simulating ordinary differential equation models in biology
Source: PLoS One. 2025 Aug 26;20(8):e0329148. doi: 10.1371/journal.pone.0329148 (PMC12380267; doi:10.1371/journal.pone.0329148)
Supplement: S1 Data — (ZIP) [file pone.0329148.s001.zip › supporting_information.pdf]

Supporting information

S1 Listing. IR generated for the Extended SIR model


```
{
  {
  "metadata": {
    "name": "",
    "type": "ode",
    "start_time": 0.0,
    "delta_time": 0.01,
    "end_time": 50.0,
    "positions": {
      "E": {
        "x": 203.0,
        "y": 870.0
      },
      "E eq": {
        "x": 1159.0,
        "y": 978.0
      },
      "E ode": {
        "x": 1310.0,
        "y": 984.0
      },
      "I": {
        "x": 190.0,
        "y": 355.0
      },
      "I death": {
        "x": 969.0,
        "y": 445.0
      },
      "I eq": {
        "x": 1230.0,
        "y": 509.0
      },
      "I ode": {
        "x": 1543.0,
        "y": 599.0
      },
      "Pathogen death": {
        "x": 930.0,
        "y": 884.0
      },
      "R": {
        "x": 187.0,
        "y": 712.0
      },
      "R death": {
        "x": 547.0,
        "y": 726.0
      },
      "R eq": {
        "x": 944.0,
        "y": 691.0
      },
      "R ode": {
```

```
    "x": 1084.0,
    "y": 727.0
  },
  "S": {
    "x": 182.0,
    "y": 204.0
  },
  "S eq": {
    "x": 889.0,
    "y": 15.0
  },
  "S ode": {
    "x": 1231.0,
    "y": 113.0
  },
  "S_death": {
    "x": 493.0,
    "y": -135.0
  },
  "Transmission": {
    "x": 516.0,
    "y": 966.0
  },
  "b": {
    "x": 669.0,
    "y": -82.0
  },
  "bd": {
    "x": 203.0,
    "y": 86.0
  },
  "be": {
    "x": 186.0,
    "y": 538.0
  },
  "c": {
    "x": 691.0,
    "y": 843.0
  },
  "g": {
    "x": 847.0,
    "y": 265.0
  },
  "infection": {
    "x": 562.0,
    "y": 181.0
  },
  "infection_free_pathogen": {
    "x": 530.0,
    "y": 484.0
  },
  "n": {
    "x": 205.0,
    "y": -42.0
  },
  "p": {
    "x": 225.0,
```

```
      "y": 1026.0
    },
    "recover": {
      "x": 1105.0,
      "y": 306.0
    }
  },
  "extension_files": [
  ]
},
"arguments": [
  {
    "name": "S_death",
    "operation": "*",
    "style": "Infixed",
    "composition": [
      {
        "name": "n",
        "contribution": "+"
      },
      {
        "name": "S",
        "contribution": "+"
      }
    ]
  },
  {
    "name": "infection",
    "operation": "*",
    "style": "Infixed",
    "composition": [
      {
        "name": "bd",
        "contribution": "+"
      },
      {
        "name": "S",
        "contribution": "+"
      },
      {
        "name": "I",
        "contribution": "+"
      }
    ]
  },
  {
    "name": "c",
    "value": 1.0
  },
  {
    "name": "I death",
    "operation": "*",
    "style": "Infixed",
    "composition": [
      {
        "name": "n",
        "contribution": "+"
```

```
    },
    {
      "name": "I",
      "contribution": "+"
    }
  ]
},
{
  "name": "bd",
  "value": 0.01
},
{
  "name": "E eq",
  "operation": "+",
  "style": "Infixed",
  "composition": [
    {
      "name": "Transmission",
      "contribution": "+"
    },
    {
      "name": "Pathogen death",
      "contribution": "-"
    }
  ]
},
{
  "name": "p",
  "value": 0.1
},
{
  "name": "Pathogen death",
  "operation": "*",
  "style": "Infixed",
  "composition": [
    {
      "name": "c",
      "contribution": "+"
    },
    {
      "name": "E",
      "contribution": "+"
    }
  ]
},
{
  "name": "S",
  "value": 1000.0
},
{
  "name": "R",
  "value": 0.0
},
{
  "name": "R death",
  "operation": "*",
  "style": "Infixed",
```

```
  "composition": [
    {
      "name": "n",
      "contribution": "+"
    },
    {
      "name": "R",
      "contribution": "+"
    }
  ]
},
{
  "name": "Transmission",
  "operation": "*",
  "style": "Infixed",
  "composition": [
    {
      "name": "p",
      "contribution": "+"
    },
    {
      "name": "I",
      "contribution": "+"
    }
  ]
},
{
  "name": "infection_free_pathogen",
  "operation": "*",
  "style": "Infixed",
  "composition": [
    {
      "name": "be",
      "contribution": "+"
    },
    {
      "name": "S",
      "contribution": "+"
    },
    {
      "name": "E",
      "contribution": "+"
    }
  ]
},
{
  "name": "E",
  "value": 0.0
},
{
  "name": "S eq",
  "operation": "+",
  "style": "Infixed",
  "composition": [
    {
      "name": "b",
      "contribution": "+"
```

```
    },
    {
      "name": "infection",
      "contribution": "-"
    },
    {
      "name": "infection_free_pathogen",
      "contribution": "-"
    },
    {
      "name": "S_death",
      "contribution": "-"
    }
  ]
},
{
  "name": "be",
  "value": 0.01
},
{
  "name": "g",
  "value": 6.0
},
{
  "name": "R eq",
  "operation": "+",
  "style": "Infixed",
  "composition": [
    {
      "name": "recover",
      "contribution": "+"
    },
    {
      "name": "R death",
      "contribution": "-"
    }
  ]
},
{
  "name": "n",
  "value": 0.1
},
{
  "name": "I",
  "value": 1.0
},
{
  "name": "b",
  "value": 100.0
},
{
  "name": "I eq",
  "operation": "+",
  "style": "Infixed",
  "composition": [
    {
      "name": "infection",
```

```
        "contribution": "+"
      },
      {
        "name": "infection_free_pathogen",
        "contribution": "+"
      },
      {
        "name": "recover",
        "contribution": "-"
      },
      {
        "name": "I death",
        "contribution": "-"
      }
    ]
  },
  {
    "name": "recover",
    "operation": "*",
    "style": "Infixed",
    "composition": [
      {
        "name": "g",
        "contribution": "+"
      },
      {
        "name": "I",
        "contribution": "+"
      }
    ]
  }
],
"equations": [
  {
    "name": "S ode",
    "operates_on": "S",
    "argument": "S eq",
    "contribution": "+"
  },
  {
    "name": "E ode",
    "operates_on": "E",
    "argument": "E eq",
    "contribution": "+"
  },
  {
    "name": "I ode",
    "operates_on": "I",
    "argument": "I eq",
    "contribution": "+"
  },
  {
    "name": "R ode",
    "operates_on": "R",
    "argument": "R eq",
    "contribution": "+"
  }
```

```
  ]
}
```

S2 Listing. Python code generated for the Extended SIR model

```
import argparse, contextlib, sys, os
import scipy
import numpy as np
np.seterr(divide="raise")

def initial_values() -> np.ndarray:
    E_0 = 0.0
    I_0 = 1.0
    R_0 = 0.0
    S_0 = 1000.0
    return np.array((
        E_0,
        I_0,
        R_0,
        S_0,
        ))

def constants() -> list:
    b = 100.0
    bd = 0.01
    be = 0.01
    c = 1.0
    g = 6.0
    n = 0.1
    p = 0.1
    return [
        b,
        bd,
        be,
        c,
        g,
        n,
        p,
        ]

def constants_with_names() -> list:
    constants_list = [
        ("b", 100.0),
        ("bd", 0.01),
        ("be", 0.01),
        ("c", 1.0),
        ("g", 6.0),
        ("n", 0.1),
        ("p", 0.1),
        ]
    return constants_list

def variable_names() -> list[str]:
    return [
        "E",
        "I",
        "R",
```

```
        "S",
        ]

def system(t: np.float64, y: np.ndarray, *constants) -> np.ndarray:
    # populations
    E,I,R,S, = y
    # constants
    b,bd,be,c,g,n,p, = constants

    dE_dt = (p*I )+- (c*E )
    dI_dt = (bd*S*I )+(be*S*E )+- (g*I )+- (n*I )
    dR_dt = (g*I )+- (n*R )
    dS_dt = b+- (bd*S*I )+- (be*S*E )+- (n*S )

    return np.array([dE_dt,dI_dt,dR_dt,dS_dt])

# includes! "ode-support.py"

def simulation_output_to_csv(sim_steps, simulation_output, write_to):
    if not simulation_output.success:
        print(simulation_output.message)
        return

    populatio_values_per_dt = simulation_output.y.T

    write_to.write(f"t,{','.join(variable_names())}\n")

    for dt, y in zip(sim_steps, populatio_values_per_dt):
        write_to.write(f"{dt},")
        write_to.write(",".join(f"{val:.4f}" for val in y))
        write_to.write("\n")

COLORS = [
    'tab:blue',
    'tab:orange',
    'tab:green',
    'tab:red',
    'tab:purple',
    'tab:brown',
    'tab:pink',
    'tab:gray',
    'tab:olive',
    'tab:cyan',
]

def plot_simulation(sim_steps, simulation_output, filename, x_label="time
     (days)", y_label="conc/ml"):
    import matplotlib.pyplot as plt
    from matplotlib.backends.backend_pdf import PdfPages

    with PdfPages(filename) as pdf:
        # All
        all_fig, all_ax = plt.subplots()
        all_fig.set_size_inches(8, 6)
        all_ax.set(title="", xlabel=x_label, ylabel=y_label)

        # Individually
```

```
        for i, (variable_name, variable_line_data) in
             enumerate(zip(variable_names(), simulation_output.y)):
            fig, ax = plt.subplots()
            fig.set_size_inches(8, 6)
            ax.set(
                title=variable_name,
                xlabel=x_label,
                ylabel=y_label,
            )
            ax.plot(simulation_output.t, variable_line_data, color=COLORS[i
                 % len(COLORS)])
            all_ax.plot(simulation_output.t, variable_line_data)

            pdf.savefig(fig)
        all_ax.legend(variable_names(),loc="best")
        pdf.savefig(all_fig)


def file_or_stdout(filename: str | None):
    if filename:
        return open(filename, 'w')
    else:
        return sys.stdout

def update_constants_with_params(constants, params):
    updated_constants = constants.copy()

    constant_names = [constant[0] for constant in constants]

    for name, value in params.items():
        for idx, (const_name, const_value) in enumerate(updated_constants):
            if const_name == name:
                updated_constants[idx] = (const_name, value)

    return updated_constants


def simulate(filename, st=0, tf=50, dt=0.1, plot=False, x_label="time
     (days)", y_label="conc/ml", params={}):
    sim_steps = np.arange(st, tf + dt, dt)

    constants_values = [value for _, value in
         update_constants_with_params(constants_with_names(), params)]

    simulation_output = scipy.integrate.solve_ivp(
        fun=system,
        t_span=(st, tf + dt * 2),
        y0=initial_values(),
        args=tuple(constants_values),
        t_eval=sim_steps,
    )

    if plot:
        plot_simulation(sim_steps, simulation_output, filename, x_label,
             y_label)
    else:
        with file_or_stdout(filename) as f:
```

```
            simulation_output_to_csv(sim_steps, simulation_output, f)

if __name__ == "__main__":
    parser = argparse.ArgumentParser()
    parser.add_argument("--st", type=float, default=0)
    parser.add_argument("--tf", type=float, default=50)
    parser.add_argument("--dt", type=float, default=0.01)
    parser.add_argument("-o", "--output", default=None)
    parser.add_argument("--csv", action=argparse.BooleanOptionalAction)
    parser.add_argument("--xlabel", type=str, default="time (days)")
    parser.add_argument("--ylabel", type=str, default="conc/ml")
    parser.add_argument("--params", type=str, default="")

    args = parser.parse_args()

    if args.params:
        params = {k: float(v) for k, v in (param.split('=') for param in
             args.params.split())}
    else:
        params = {}

    simulate(
        args.output,
        plot=not args.csv,
        st=args.st,
        tf=args.tf,
        dt=args.dt,
        x_label=args.xlabel,
        y_label=args.ylabel,
        params=params
    )
```
